# Supplementary material for: Increase Paclitaxel Sensitivity to Better Suppress Serous Epithelial Ovarian Cancer via Ablating Androgen Receptor/Aryl Hydrocarbon Receptor-ABCG2 Axis
Source: Cancers (Basel). 2019 Apr 2;11(4):463. doi: 10.3390/cancers11040463 (PMC6521308; doi:10.3390/cancers11040463)
Supplement: Supplementary file 1 [file cancers-11-00463-s001.pdf]

Supplementary information

Supplementary Tables

Table S1. The qRT-PCR primer sequences for mRNA expression

Primer name	sequences
AR	F: 5'-TgTCCATCTTgTCgTCTTC-3'<br>R: 5'-CCTCTCCTTCCTCCTgTAg-3'
AhR	F: 5'-CCACTTCAgCCACCATCCAT-3'<br>R: 5'-AAgCAggCgTgCATTAgACT-3'
ABCG2	F: 5'-gggTTCTCTTCTTCCTgACgACC-3'<br>R: 5'-TggTTgTgA gATTgACCAACAgACC-3'

Table S2. The PCR primers sequencing for ABCG2 promoter region amplification

Primer name	location	sequences
Full length	-3268/+362	F:5'gAgCTCgCAgCCTTgAACCCCggggCTCAAgC3'<br>R:5'gggAAgCTTgCACggTgCgTTCCTAAATCCTACCC3'
1F1R	-3268/-1396	F:5'gAgCTCgCAgCCTTgAACCCCggggCTCAAgC3'<br>R:5'gggAAgCTTggCCgTTAATgACTgTTTgCAAAAACT3'
2F4R	-1396/+362	F:5'gAgCTCAgTTTTTgCAAACAgTCATTAACggCC3'<br>R:5'gggAAgCTTgCACggTgCgTTCCTAAATCCTACCC3'
3F4R	-959/+362	F:5'gAgCTCAATTTTACCCAAATgTTTgggCgCA3'<br>R:5'gggAAgCTTgCACggTgCgTTCCTAAATCCTACCC3'
4F4R	-523/+362	F:5'gAgCTCTTCATTCAgTAAgTTTCTCCCCTTTCC3'<br>R:5'gggAAgCTTgCACggTgCgTTCCTAAATCCTACCC3'

Table S3. The qRT-PCR primer sequences for ChIP assay

Primer name	sequences
ChIPR1.2F	F: 5'-gACTTggAACCAACCCAAATg-3'
ChIPR1.2R	R: 5'-TCCCTACAAAggACATgAACTC-3'
ChIPR2.5F	F: 5'-ggCCTTTAAgggTCTTgAAACTg-3'
ChIPR2.5R	R: 5'-TggTTCACCCAATgCAggAAA-3'
ChIPR3.4F	F: 5'-gCCTTTCCACACCATCggA-3'
ChIPR3.4R	R: 5'-AggAAAggggAgAAACTTACTg-3'

Table S4. IC_{50} of AR-manipulated EOC cells under paclitaxel treatment (* $p < 0.05$)

Cell line	Manipulation	IC_{50}
Hey A8	Vector control	0.0059 ±0.004 μM*
	hAR	0.024 ±0.019 μM*
	siAR	0.0011 ±0.0002 μM
SKOV3ip1	Vector control	0.003 ±0.002 μM*
	hAR	0.008 ±0.001 μM*
	siAR	0.0025 ±0.002 μM
OVCAR3	Vector control	0.001 μM*
	hAR	0.009 ±0.002 μM*
	siAR	0.001 μM

Table S5. IC_{50} of EOC cells treated by paclitaxel combined with ASC-J9 or MDV3100

Cell line	treatments	IC_{50}
Hey A8	Paclitaxel	0.0063 ± 0.003 μM
	Paclitaxel/ 10 μM MDV	0.0051 ± 0.0028 μM
	Paclitaxel/ 5 μM ASC-J9	0.0016 ± 0.0005 μM
SKOV3ip1	Paclitaxel	0.0058 ± 0.0007 μM
	Paclitaxel/ 10 μM MDV	0.0054 ± 0.0005 μM
	Paclitaxel/ 5 μM ASC-J9	0.002 ± 0.001 μM
OVCAR3	Paclitaxel	0.002 ± 0.001 μM
	Paclitaxel/ 10 μM MDV	0.0027 ± 0.0003 μM
	Paclitaxel/ 5 μM ASC-J9	0.0007 ± 0.00026 μM

Supplementary figures

Supplementary Figure S1. Chung et al

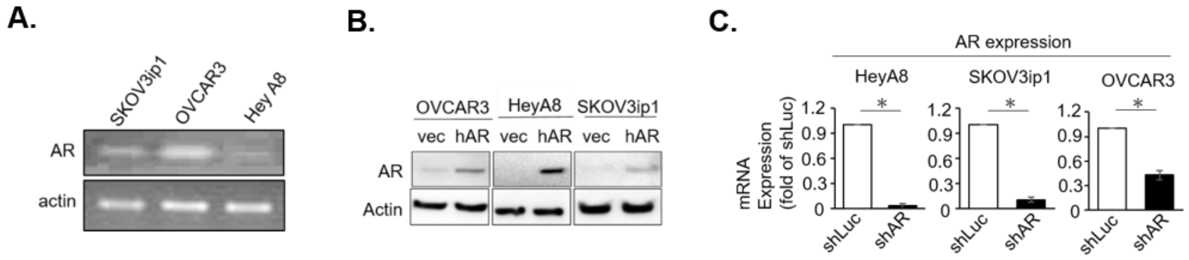

Supplementary Figure S1. AR expression and association with paclitaxel sensitivity and resistance. (A) Basal expression of AR mRNA in three serous type cells. Actin was used as an internal control. **(B)** Overexpression of AR in three EOC serous subtype cell lines. Cells were infected with either pWPI vector control or pWPI-hAR plasmids to establish AR-stable clones. Cells with AR overexpression were then lysed to perform western blotting. Total protein was loaded at 40μg/lane and AR antibody was used to detect AR expression. Actin was used as an internal control. **(C)** Cells were infected with either pLKO.1-shLuc vector control or pLKO.1-shAR plasmids to establish AR-knockdown stable clones. Knockdown of AR expression in EOC serous subtype cells. RNA was extracted and cDNA was synthesized using 5μg of total RNA. AR expression was determined using qRT-PCR. Actin was used as an internal control. Results of mRNA expression were calculated by $2^{-\Delta\Delta Ct}$ and normalized by shLuc.

ataaaagacattccatgttactacccagaacaaatgaacactttcctgctattttctttctttctctttttctttagagtggggtctcactctgatcatggctcgctgcagccttgaaccccggggctcaagc

aatccttccacctcagcccccaaatagctaggtccacaggcatgtgccatcacatccagctaattttaaaaaattgtttgtacaaatttacaagaaaaaacaaacaaccccatcaaaaagtggg

cgaaggacatgaacagacacttctcaaaagaagacatttatgcagccaaaaaacacatgaagaaatgctcatcatcactggccatcagagaaatgcaaatcaaaaccactatgagatatcatct
putative ARE

cacaccagttagaatggcaatcattaaaaagtcaggaaacaacaggtgctggagaggatgtggagaaataggaacacttttacactgttggtgggactgtaaactagttcaaccattgtggaagtc

ggtgtggcgattcctcagggatctagaactagaaataccatttgacccagccatcccattactgggtatatacccaaaggactataaatcatgctgctataaagacacatgcacacgtatgtttattgc

agacacatgcacacgtatgtttattgcggcactattcacaatagcaaagacttggaaccaacccaaatgtccaacaatgatagactggattaagaaaatgtggcacatatacaccatggaatacta
putative ARE

tgcagccataaaaaatgatgagttcatgtcctttgtagggacatggatgaaattggaaaccatcattctcagtaaactatcgcaagaacaaaaaaccaaacaccgcatattctcactcataggtggg
putative ARE putative ARE

aattgaacaatgagatcacttggacacaggaaggggaatatcacactctggggactgtggtggggtcgggggagggggggagggatagcattgggagatatacctaatgctagatgacacgttagt
putative ARE

gggtgcagcgcaccagcatggcacatgtatacatatgtaactaacctgcacaatgtgcacatgtaccctaaaacttagagtataataaaaaaaaaaaaaattaaaaaaaaaaattgtttgtagagac

aggggctccctgtgttgcctaggcttgtctcgaattcttgggctcaagcaatcctctggcctcctcccaaagtgctgggattacaggtgtaagccaccacacccagtgtaatttaaaatatttttttttttgt

agagatagggtctcagtatgttgcccaggctggtcttgaactcctgggctcaagcaattctttggcctctgcctcccaaagtgttgggattacaggagtgagctgtcactccttgcccagcaattttcta

cgtataggtttttttttttttttttttttttgagatggagtctcacgttgtcacccaggctagagtgcagtgacatgatcttggctcactgcagcctctgcctccctggctccagcaattctcctgcctcagcct

cctgggtagctgggattacaggcatgagccaccatgcccggctaattttgtatttttagtagagaagggtttcaccatgttggccaggctgctcttgaagtcctgatatcaggtgatccacctgcctcg

gcctcccaaagtactaggattacaggcgtgagccaccgtgcctggcctctatgtatagggttttgatttgtttttactttgcataattacagaatgctatattcaactaagtatccatttttaaaaatgtgctt

catatcagtttcgtgtgtgttttctccatttttgttcagtctcctcctgtagtgccttcagatcttgctggaaatgttttcatttttctggtatcataaaatttattcttttcacttatctgggtgagctaaaaaga

aaattaaaataaaatttattttcggagatttttcagtttttgcaaacagtcattaacggccaggtctgaaaactttatcttaatttttttttttttcttgagacagagtctcactctgtggcccaggctggagt

gcagtggtgggatctcgggtcatttgcaacctctgcctcctgtgttcaagcgcttcttgttccttagcctcgcgagtagctgggattacagcctcgccaccacacccagctaacttttgtatttttagtaga

ggcagggtttcaccatgttggccaggctggtctcgaactcctggcctcaagtgatctgcctgcgtcagcctcccaaagtgctgggattacaggcatgagccaccgtgctggccttaattttgtcttaaa

atgaagtatgactaacatgtatttctatatgggcctttaagggtcttgaaactgacagaaattaattcaaaagaataattttacccaaatgtttgggcgcatcatgaatatataccttaaaattcttttaaa
alternative ARE

aagcatgcggtatggtatatttaaaattctttttaaaaaattatgtatagtcaattttcttatctaggcttcctagctaaaactttcctgcattgggtgaaccattaatagttattcctagtgttgatttaaaca

acttaaatgatactatgatttttgtttgtaaatgcaaaccagaaaatatctgcatgcaggtactaatttcctagggtagatgcagcaggtagatgttgggatggctacactcactcacaaagcctgatgg

cccgtttcctgaacatgcgcaatcactttctagcctttccacaccatcggaatattgcacagaagtatgtcagtcacaggagtgtaaagaataacatttaaactatggtaatattttcattcagtaagttt
alternative ARE

ctcccctttccttccttgggttagattcagtgcctgatttttttaggccctggggcgcaagcatccactttctcagaatcccattcaccagaaaccacccatttaacttgctctgggtgcgagcagcgcttg

tgactgggcaacctgtgcgtcagcgtccccggtgcttcggcgctccggccagtgacggcgaccaaacccagctaggtcagacgaggtactgatcagcccaatgagcgcctggtgattctcgtagtt

aatcactctggttcattccgttcgatcccggaggcgggagtgtttggcttgtccctgcgtgtcacggcagggtgaccctagccccgagggagggcggtggtaccagtcctgctggcggctcagcgcg
core DRE

gcaggacacgtgtgcgctttcagccgggtcgcagggcgcttatcgcggcccggcagtcggggccacgcctcacccccgcccgcgaaccccgacctggggaaacccggggcgctggggagggg

+1
ccactgcgttcagctctggcggtccacagcccgaagcgcggcttaggaagttcgtgtcagcgctgcctgagctcgtcccctggatgtccgggtctccccaggcggccacccgccggctcccatcgt
transcriptional start site

gacctccagccgcagcgcctcccacgccggccgccgcgcgaggggagcgctcgggcgcgccgggtgtggttgggggaaggggttgtgccgcgcgcggctgcgtgctgtgcccactcaaaag

gttccgggcgcgcaggagggaagaggcagtgcccgccactcccactgagattgagagacgcggcaaggaggcagcctgtggaggaactgggtaggatttaggaacgcaccgtgcacatgcttg

gtggtcttgttaagtggaaactgctgctttagagtttgtttggaaggtccgggtgactcatcccaacatttacatccttaattgttaaagcgctgcctccgagcgcacgcatcctgagatcctgagccttt

ggttaagaccgagctctattaag

Supplementary Figure S2. Nucleotide sequences of the 5’ flanking promoter region of the human ABCG2 gene. ABCG2 promoter region nucleotide sequences were obtained from NCBI database (accession no. NC_018915). Transcription start site was indicted as +1.

Supplementary Figure S3, Chung et al.

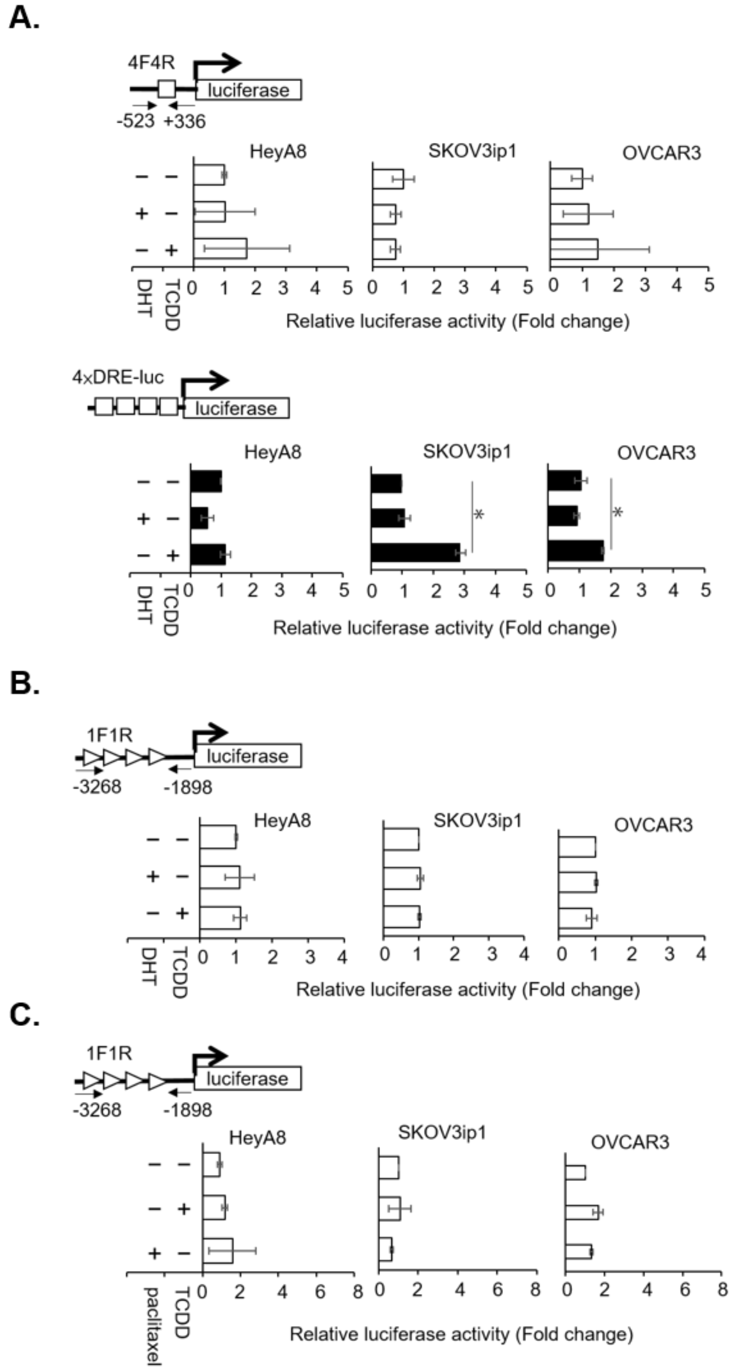

Supplementary Figure S3. Luciferase activity of the human ABCG2 5'-promoters. (A) 4F4R luciferase activity cannot be induced under TCDD or DHT treatment in EOC cells. Upper panel: 4F4R and pRL-TK were co-transfected into three serous type cells and treated

with DMSO, 10^{-8} M DHT, or 5nM TCDD. The core DRE region is indicated as □. Results were the fold changes in response to DMSO treatment. Down panel: 4xDRE-luc plasmid under the same treatments served as a positive control. **(B)** 1F1R luciferase activity cannot be induced under TCDD or DHT treatment in EOC cells. 1F1R and pRL-TK were co-transfected into three serous type cells and treated with DMSO, 10^{-8} M DHT, or 5nM TCDD. The putative ARE is indicated as ▷. Results were the fold changes in response to DMSO treatments. **(C)** 1F1R luciferase activity cannot be induced under TCDD or paclitaxel treatment in EOC cells. 1F1R and pRL-TK were co-transfected into three serous type cells and treated with DMSO, 2nM paclitaxel, or 5nM TCDD.
